# Toward the clinical application of the Child Psychosis-Risk Screening System (CPSS)

**DOI:** 10.1192/j.eurpsy.2022.585

**Published:** 2022-09-01

**Authors:** Y. Hamasaki, M. Matsuo, Y. Sakaue, R. Sanada, T. Nakayama, S. Michikoshi, S. Ueba, N. Kurimoto, T. Hikida

**Affiliations:** 1 Kyoto Women’s University, Faculty Of Contemporary Society, kyoto, Japan; 2 Shiga University of Medical Science, Department Of Psychiatry, Otsu、Shiga, Japan; 3 Shiga University of Medical Science, Department Of Pediatrics, Otsu, Shiga, Japan; 4 Saiseikai Moriyama Municipal Hospital, Department Of Pediatrics, Shiga, Japan; 5 Shigasato Hospital, Department Of Psychiatry, Shiga, Japan; 6 Osaka University, Laboratory For Advanced Brain Functions, Institute For Protein Research, Osaka, Japan

**Keywords:** Child Behavior Checklist, Psychosis, CPSS, prodrome

## Abstract

**Introduction:**

In our previous study, we have developed the Child Psychosis-risk Screening System (CPSS), which incorporates psychological and behavioral characteristics of childhood into an algorithm, based on a retrospective survey.

**Objectives:**

In this study, we actually tried to evaluate the risk of psychosis in pediatric and psychiatric outpatients using the CPSS.

**Methods:**

We conducted an epidemiological study of 323 outpatients aged 6-18 years visiting pediatric and psychiatric departments using CBCL and clinical data (sex, age, winter birth, chief complaint, diagnosis, abuse, bullying, hikikomori). ROC analysis was used to assess the accuracy of CPSS predictions. Cross-sectional logistic regression analysis was performed on the clinical data to identify factors associated with risk groups exceeding the cutoff value.

**Results:**

The results of the ROC analysis showed that the AUC (Area under the ROC Curve) was 80.3%, indicating that the CPSS has Moderate accuracy. The cutoff value was 98.11% (sensitivity: 0.857, specificity: 0.835), and 18% of the subjects were identified as risk groups above this value. Cross-sectional logistic regression analysis showed that schizophrenia diagnosis, no abuse, winter birth, and hikikomori were associated with the risk group, with respective odds ratios of 22.88, 10.76, 1.91, and 1.37.

**Conclusions:**

The results of this study suggest that the CPSS can be applied to pediatric practice for early detection of risk for psychosis. The risk group is also present among pediatric patients with physical chief complaints. The factors suggested to be associated with risk groups may reflect the factors acting on the critical period of psychosis onset and the dynamic state.

**Disclosure:**

No significant relationships.

